# Behind the Counter

**Published:** 1893-01

**Authors:** 


### BEHIND THE COUNTER.

No. 1.

It is with reluctance that, in redeeming my promise to give the readers of HALL'S JOURNAL OF HEALTH some short paper on a worn and threadbare subject, I find it necessary to speak of myself, in view of what is to follow, growing out of my experiences as a saleslady in one of our mammoth dry goods stores.

Having been deprived of both father and mother at an early age, I was fortunate in finding a home with near relatives, who gave me welcome, and strove in every kindly way to make me forgetful of my orphanage, being continued at one of our better class schools with scarcely an interruption up to the period of my graduation, I need not say how long ago, suffice it, that measured by the common standard, I had acquired a fairly good education.

My uncle, during this period, was possessed of ample means, and provided his home with all the more solid comforts, with no lavish display merely of ornamental embellishments, of which he complained as being always in the way. But oil paintings and statuary if done after his own notion of high art, were exceptions. He had at this time a great many warm friends who were anxious to add to his ample store, by some sort of partnership wherein my uncle was to furnish the capital. Some of these schemes were put in operation, and he was advised from time to time of the state of affairs. Strange to say, they were always on the very point of expectation. A little more capital and success would be assured. My uncle was credulous to a fault, and not until his bank account dwindled to a condition which demanded a curtailment of family expenses, did he undertake to follow his investments, a no easy task.

When I was made aware of this change in his affairs, I determined, in spite of much friendly opposition, to become self-supporting, and at once set about its accomplishment.

I shall never forget the feelings which possessed me, when on a cool November morning, quite alone, I timidly entered "our store," as prearranged, and reported for duty, an ordeal I had gladly escaped, but *duty*, as I conceived it, was the spur of resolution, and I passed through it as bravely as could be expected of one so strange to the world of traffic as was I.

It was business, nothing but business from the start. The superintendent, to whom I was directed, looked me over hastily, much as he would regard some new zoölogical importation, and with a stereotype drawl, remarked, "Well, young woman, what are you capable of ; what position can you fill with us?" On learning that this was my first assay at store keeping, my inquisitor gave a kind of protesting shrug of the shoulders, and bidding me follow him, led the way to the basement, and with a few words of explanation, turned me over to a rather curious female in the meridian of life, who received me not unkindly, and conducting me to a little partially lighted room common to the store hands, assigned me a peg for my cloak and hat with the assurance that that particular peg was to be solely *mine*, so long as I was fortunate enough to hold my new position. Then followed my initiation into the mysteries of kitchen utensils, over which my superior presided as saleswoman, my part in the business being that of a junior assistant, in which capacity I acquitted myself as well as could be expected of a raw hand. This was the comment of my superior at the close of my first day, and I returned to my home after darkness had fairly set in, weary enough, but by no means shaken in my resolution to make my way in life by my unaided endeavors. PAULINE.

(*To be continued.*)

